# Analysis of terpenoids and their gene regulatory networks on the basis of the transcriptome and metabolome of *Opisthopappus longilobus*

**DOI:** 10.3389/fpls.2022.1015942

**Published:** 2022-09-21

**Authors:** Hua Liu, Yuhong Chai, Haixia Chen, Wendan Chen, Yushu Li, Wenchao Liu, Shuang Guo, Lei Sun, Xiumei Zhou, Conglin Huang, Xiaowei Tang, Chang Luo, Dongliang Chen, Xi Cheng

**Affiliations:** ^1^Institute of Grassland, Flowers and Ecology, Beijing Academy of Agriculture and Forestry Sciences, Beijing, China; ^2^School of Horticulture and Landscape Architecture, Henan Institute of Science and Technology, Xinxiang, China; ^3^Beijing Vocational College of Agriculture, Beijing, China; ^4^Beijing Liu Wenchao Institute of Summer Chrysanthemum Breeding Science and Technology, Beijing, China

**Keywords:** *Opisthopappus longilobus*, transcriptome, metabolome, aroma, terpenoid metabolites

## Abstract

*Opisthopappus longilobus*, which is a unique wild plant resource in China, produces leaves and flowers with distinct aromas. However, there have been relatively few molecular studies on its floral aroma, which has hindered the research on this plant species at the molecular level and the breeding of novel varieties. In this study, transcriptome and metabolome analyses were performed using *O. longilobus* leaves, buds, and inflorescences at the exposure, initial opening, and blooming stages. Using high-quality reads and assembly software, a total of 45,674 unigenes were annotated according to the Nr, Swiss-Prot, KOG, and KEGG databases. Additionally, a GC-MS system and a self-built database were used to detect 1,371 metabolites in the leaves, buds, and inflorescences. Terpene metabolites were the most common compounds (308 in total). We analyzed the gene network regulating terpenoid accumulation in *O. longilobus* and identified 56 candidate genes related to terpenoid synthesis. The expression of *OlPMK2*, *OlMVK1*, *OlTPS1*, and *OlTPS3* may lead to the accumulation of 11 different terpenoids specifically in the inflorescences at the exposure, initial opening, and blooming stages. The generated data may be useful for future research on *O. longilobus* genetic resources and the molecular mechanism regulating aroma formation in this plant species. The findings of this study may be used to accelerate the breeding of new *O. longilobus* varieties with enhanced aromatic traits.

## Introduction

*Opisthopappus longilobus* Shih is a perennial herb belonging to the genus *Opisthopappus* (family Compositae), which is endemic to China and includes two species, namely *Opisthopappus taihangensis* and *O. longilobus* (Wang and Yan, 2013; Wang and Yan, 2014; Chai et al., 2018). The whole *O. longilobus* plant is fragrant because of the substantial abundance of aromatic oil. Moreover, from June to September (i.e., the flowering period), it produces white flowers (Guo et al., 2013; Wang, 2013; Jia and Wang, 2015; Ye et al., 2021). Many recent studies on *O. taihangensis* and *O. longilobus* focused on genetic diversity and genetic structure, tissue culture, community characteristics, and antioxidant and antibacterial compounds (Guo et al., 2013; Chai et al., 2018; Zhang et al., 2018; Ye et al., 2021). The leaves and inflorescences of *O. longilobus* have special aromas that are distinct from the aromas of most chrysanthemum species (Guo et al., 2020). Additionally, its essential oil has antibacterial, anti-inflammatory, antioxidant, and other medicinal effects (Zhang et al., 2018; Guimaraes et al., 2019). Previous research confirmed that *O. longilobus* is a rich source of aromatic oils and inulin as well as sesquiterpene lactones with beneficial effects on the heart and anti-cancer, anti-insect, and analgesic effects (Zeng et al., 2010). Because *O. longilobus* flowers that are steamed and then dried in darkness can be used to improve liver health and vision, while also decreasing fevers and eliminating thirst, people living in the Taihang Mountain region often collect *O. longilobus* flowers and use them to produce beverages with health benefits (Ye et al., 2021). Accordingly, the mechanisms underlying the synthesis of aromatic substances in *O. longilobus* should be thoroughly investigated.

Aromatic substances in plants can be categorized as terpenes, aromatic compounds (e.g., phenylpropane), and fatty acid derivatives according to their biosynthetic pathways, which involve reactions catalyzed by various enzymes (Nagegowda, 2010). Terpenes, which are the most diverse class of organic compounds in nature, are important for the formation of floral aromas (Wong et al., 2017; Zhang et al., 2021). They can be divided into semi-terpenes, monoterpenes, sesquiterpenes, diterpenes, triterpenes, tetraterpenes, polyterpenes, and irregular terpenes (He et al., 2020). Terpenes consist of several isoprene (C5) units, and they are synthesized by a complex network regulated by multiple genes and enzymes (Zhong et al., 2022).

Terpenes share common precursor substances, including isopentenyl pyrophosphate and dimethylallyl pyrophosphate (Nagegowda, 2010). There are two different metabolic pathways for their biosynthesis. One is the mevalonate (MVA) pathway in the cytoplasm and mitochondria, wherein it is involved in the biosynthesis of secondary metabolites, including sterols, sesquiterpenes, and triterpenes. Another is the plastid-based 2-C-methyl-D-erythritol 4-phosphate (MEP) pathway, which is mainly involved in the biosynthesis of monoterpenes, diterpenes, and carotenoids (Nagegowda, 2010; Zhong et al., 2022). There has been considerable progress in the research on the biochemical pathways and molecular mechanisms regulating floral volatiles (Wong et al., 2017; Fan et al., 2018; Ramya et al., 2018), but because of the lack of relevant data, the metabolism of aromatic substances in *O. longilobus* leaves and inflorescences remains a mystery. The strong aromas of its flowers and leaves make *O. longilobus* a very valuable aromatic plant resource in various industries (e.g., agriculture, medicine, perfume, and cosmetics) (Zhang et al., 2018). Therefore, the metabolic mechanism regulating the aromatic substances in *O. longilobus* must be elucidated.

There are currently relatively few molecular studies related to *O. longilobus* aromas. More specifically, the lack of transcriptome and metabolome information is a major obstacle for molecular research on this plant species as well as the breeding of novel varieties with enhanced traits (Chai et al., 2018; Ye et al., 2021). Thus, we used bioinformatics-based methods to perform transcriptome and metabolome analyses of *O. longilobus* leaves, buds, and inflorescences at the exposure, initial opening, and blooming stages. We thoroughly investigated the compositions of aromatic substances in the leaves and inflorescences and the genes in the associated regulatory pathways, while also analyzing the diversity in the compositions and contents of terpenoids and other metabolites in the leaves and inflorescences at different developmental stages. Additionally, we explored the gene regulatory network controlling terpenoid contents and extracted the essential oil from leaves for a subsequent analysis of its compositions and contents. The results of this study provide researchers with the theoretical basis for clarifying the molecular mechanism regulating aroma formation in *O. longilobus.* The information provided herein may also be relevant for breeding *O. longilobus* as well as for conserving and developing *O. longilobus* resources.

## Materials and methods

### Plant materials and RNA extraction

**Figure 1:**
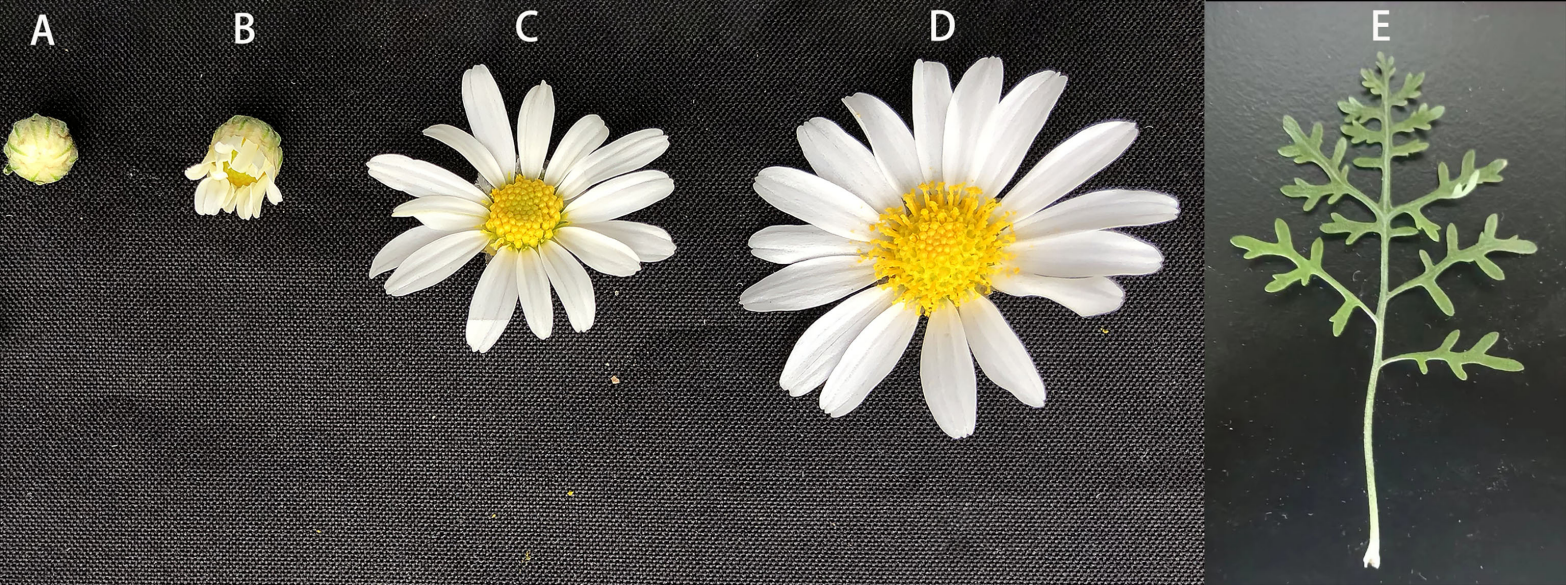
*Opisthopappus longilobus* samples used for the transcriptome and metabolome analyses. **(A–E)** Bud, inflorescences at the exposure, initial opening, and blooming stages, and a leaf, respectively.

The *O. longilobus* plants used in this study were obtained and propagated from cuttings and then cultivated in a greenhouse at the Shunyi Base of the Beijing Liu Wenchao Institute of Summer Chrysanthemum Breeding Science and Technology (116.3°E, 40.0°N). The plants started to flower approximately 90–120 days after they were transplanted. About 10–20 buds and inflorescences at the exposure, initial opening, and blooming stages as well as leaves were collected from *O. longilobus* plants from 7 am to 10 am, with three biological replicates per sample (**Figure 1**). The collected *O. longilobus* materials were immediately frozen in liquid nitrogen and then stored at −80°C. After grinding the frozen materials to a powder, total RNA was extracted using the RNeasy Plant Mini Kit (Qiagen, Shanghai, China). The quality of the extracted RNA was assessed using the NanoDrop ND2000 Spectrophotometer (Thermo Scientific, Waltham, MA, USA). The *O. longilobus* plant samples were used for the transcriptome and metabolome analyses.

### Sample preparation and GC-MS analysis

Frozen samples were ground to a powder, centrifuged, and mixed evenly by shaking. For each sample, approximately 0.5 g ground material was added to 1 mL sterile water, after which the solution was added to a headspace flask. A saturated NaCl solution and 10 μL (50 μg/mL) internal standard solution were then added to the flask. Finally, the samples were used for the headspace solid phase microextraction (HS-SPME) and gas chromatography-mass spectrometry (GC-MS) analysis. The HS-SPME was performed at 60°C, with a 5-min oscillation. The 120 µm DVB/CWR/PDMS extractor head was inserted into the headspace flask for 15 min. The samples were resolved at 250°C for 5 min and then separated and identified by GC-MS. The extractor heads were aged in a Fiber Conditioning Station at 250°C for 5 min before sampling. The GC analysis was completed using the DB-5MS capillary column (30 m × 0.25 mm × 0.25 µm; Agilent J&W Scientific, Folsom, CA, USA) and highly pure helium (purity not less than 99.999%) as the carrier gas. The constant flow rate was 1.2 mL/min. The inlet temperature was 250°C, and a split-less injection was performed, with a solvent delay of 3.5 min. The temperature was kept at 40°C for 3.5 min, then increased to 100°C at 10°C/min, then increased to 180°C at 7°C/min, and finally increased to 280°C at 25°C/min for 5 min. The MS conditions were as follows: electron bombardment ion source; ion source temperature, 230°C; four-stage rod temperature, 150°C; mass spectrum interface temperature, 280°C; electron energy, 70 eV; scanning mode, selected ion detection mode; and qualitative and quantitative ion accurate scanning (GB 23200.8-2016).

### Screening for differentially accumulated metabolites

An orthogonal partial least squares discriminant analysis (OPLS-DA) was performed using the OPLSR.Anal function of the MetaboAnalystR package in the R software. On the basis of the OPLS-DA results, the Variable Importance in Projection (VIP) value of the OPLS-DA model obtained *via* the multivariate analysis was used to preliminarily identify the DAMs between species or tissues. The P-value and the fold-change for the univariate analysis were combined to screen for DAMs. The screening criteria were as follows: VIP ≥ 1 and fold-change ≥ 2 or ≤ 0.5.

### Illumina sequencing and data processing

The mRNA in the total RNA was enriched using oligo-(dT) beads and then fragmented in fragmentation buffer before being reverse transcribed into single-stranded cDNA using random primers. After synthesizing the second strand, the cDNA was purified using the QiaQuick PCR Purification Kit. A poly-A tail and an Illumina sequencing adapter were added to the purified cDNA. The cDNA fragments were separated by agarose gel electrophoresis and then appropriate fragments were selected for a PCR amplification. The subsequent transcriptome sequencing (RNA-seq) analysis was performed using the Illumina HiSeq™ 4000 system at Gene Denovo Biotechnology Co. (Guangzhou, China). The raw sequencing data were strictly filtered to obtain high-quality clean reads. The *de novo* transcriptome assembly was performed using Trinity software to link overlapping reads to form longer fragments, of which the N-free fragments were assembled into unigenes.

### Gene expression analysis by quantitative real-time polymerase chain reaction (qRT-PCR)

To verify the RNA-seq data, a qRT-PCR analysis was performed to calculate the relative transcription levels of 18 genes. Total RNA was extracted from *O. longilobus* leaves at the flowering stage and from inflorescences at different developmental stages. The extracted RNA was treated with DNase (Promega, Wisconsin, USA) and then cDNA was synthesized using the HiScript III All-in-one RT SuperMix Perfect for qPCR (Vazyme, Nanjing, China). The qRT-PCR analysis was completed using the PikoReal Real-Time PCR system (Thermo Fisher Scientific, Germany). For each 20-μL reaction volume, 2 μL cDNA was added as the template. The PCR conditions were as follows: 95°C for 5 s; 39 cycles of 54°C for 30 s and 72°C for 30 s. Relative gene expression levels were determined using gene-specific primers (**Supplementary File 1**). Three biological replicates were analyzed for all samples. The chrysanthemum protein phosphatase 2A gene was used as the reference gene (Liu et al., 2021b). Relative gene expression levels were calculated according to the 2^−ΔΔCt^ method (Livak and Schmittgen, 2001).

### Extraction of essential oil

*Opisthopappus longilobus* leaves were harvested during the vegetative growth period and then added to Desktop Essential Oil Pure Water Distillers (Luosha Biological Company, Jiangsu, China). Distilled water was added for a solid:liquid ratio of 1:2 for the extraction of essential oil. After the distillation, the essential oil was collected and examined in terms of the color and quality. Additionally, the essential oil extraction rate was calculated. The samples were kept at 4°C before analyzing their compositions or they were maintained at −80°C for long-term storage.

### Integrated analysis of the transcriptome and metabolome data

To evaluate the potential correlation between gene expression levels and metabolite contents, a Pearson correlation test was performed to analyze gene expression and metabolite contents. Significant correlations were determined using the following criteria: a Pearson correlation coefficient (PCC) > 0.8 and P < 0.05 (Li et al., 2021a; Wang et al., 2021a; Zhan et al., 2022). They were then visualized using the Cytoscape software (version 3.7.2) (Cytoscape Consortium, San Diego, CA). Additionally, the differentially expressed genes (DEGs) and DAMs in the terpenoid biosynthesis pathway were mapped to Kyoto Encyclopedia of Genes and Genomes (KEGG) pathways.

### Statistical analysis

Three biological replicates were used for the GC-MS, RNA-seq, and qRT-PCR analyses. Significant differences in gene expression were determined on the basis of the following thresholds: FDR < 0.05 and |log_2_(fold-change)| > 1. Only DEGs with at least a 2-fold change in expression were used for the DEG analysis. The direction of gene sequences and protein functions were determined using the blastx algorithm (http://www.ncbi.nlm.nih.gov/BLAST/), with an E-value threshold of 1e-5. The unigene sequences were aligned to the sequences in the NCBI non-redundant protein (Nr) database (http://www.ncbi.nlm.nih.gov), the Swiss-Prot protein database (http://www.expasy.ch/sprot), the KEGG database (http://www.genome.jp/KEGG), and the eukaryotic homologous groups (KOG) database (http://www.ncbi.nlm.nih.gov/KOG). The top 20 unigenes with at least 33 high-scoring pairs were selected for the Gene Ontology (GO) functional annotation using the Blast2GO software. Individual genes were functionally classified using the WEGO software.

## Results

### Total metabolite analysis

**Figure 2:**
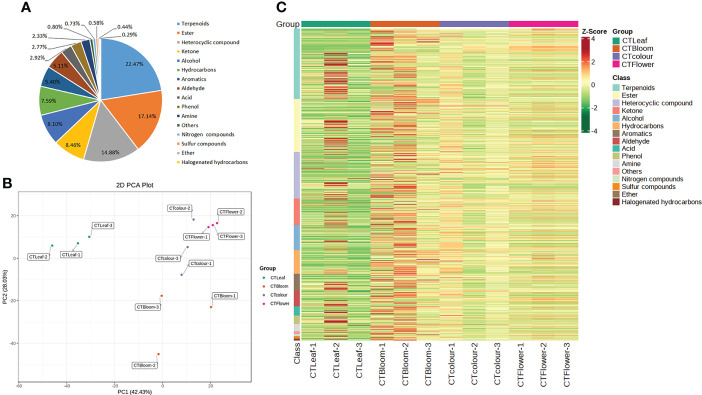
Metabolites detected in *O. longilobus.* **(A)** Classes and proportions of the metabolites detected in *O. longilobus.* **(B)** Results of the principal component analysis of the *O. longilobus* metabolome. **(C)** Heat map of the metabolites in different *O. longilobus* sample groups. Sample names are provided on the horizontal axis, whereas the metabolite information is provided on the vertical axis. The different colors for the Z-score represent relative contents (i.e., red and green reflect high and low contents, respectively).

Using the GC-MS platform and a self-built database, we detected 1,371 metabolites in the *O. longilobus* leaves and inflorescences. More specifically, 1,243, 1,338, 1,296, and 1,337 metabolites were detected in the leaves and inflorescences at the exposure, initial opening, and blooming stages, respectively. There was considerable diversity in the types of metabolites that were identified (**Supplementary File 2**). The classification of these metabolites revealed that the most common metabolites were terpenoids (308), followed by esters (235), heterocyclic compounds (204), ketones (116), alcohols (111), hydrocarbons (104), aromatic hydrocarbons (74), aldehydes (70), acids (40), phenols (38), amines (32), unidentified compounds (11), nitrogenous compounds (10), sulfur compounds (8), ethers (6), and halogenated hydrocarbons (4) (**Figure 2A**).

We performed a principal component analysis of the *O. longilobus* metabolome to detect the metabolic differences between the samples in each group and the changes within the group as a whole. The first and second principal components (PC1 and PC2) accounted for 42.43% and 28.03% of the total variance, respectively (**Figure 2B**). The accumulation of aromatic substances in the *O. longilobus* inflorescences varied among the examined developmental stages. The leaves were clearly separated from the inflorescences at different developmental stages, indicative of the considerable metabolic differences among the samples. The cluster heat map of the metabolites among different *O. longilobus* sample groups (**Figure 2C**) indicated there were clear differences in the accumulation of metabolites between the inflorescences at different developmental stages as well as between the leaves and inflorescences at different developmental stages. Hence, clarifying the changes in metabolite contents is important for elucidating the mechanism underlying the aroma formation in *O. longilobus* leaves and inflorescences.

We analyzed the terpenoids in *O. longilobus* leaves and inflorescences at different developmental stages. A total of 293, 302, 293, and 301 terpenoids were detected in the leaves and the inflorescences at the exposure, initial opening, and blooming stages, respectively (**Supplementary File 3**). A comparison with the *O. longilobus* leaves indicated 11 terpenoids were specific to the inflorescences (e.g., 2-cyclohexen-1-one, 2-hydroxy-3-methyl-6-(1-methylethyl)-; squamulosone; and 6,7-dimethyl-1,2,3,5,8,8a-hexahydronaphthalene). In contrast, six terpenoids were specific to the leaves (e.g., hibaene;

1,1,7,7a-tetramethyl-1a,2,6,7,7a,7b-hexahydro-1H-cyclopropa[a]naphthalene; and aromandendrene). The following four terpenoids were specific to the inflorescences at the exposure and blooming stages: bicyclo[7.2.0]undec-4-ene, 4,11,11-trimethyl-8-methylene-, [1R-(1R*,4Z,9S*)]-; cyclohexanol, 1-methyl-4-(1-methylethyl)-, cis-; cyclopropanecarboxylic acid, 2,2-dimethyl-3-(2-methyl-1-propenyl)-, (1R-trans)-; and bornyl acetate. There were no terpenoids that were exclusive to the inflorescences at the initial opening stage. These findings suggested that the types of terpenoids in the *O. longilobus* inflorescences did not change substantially between the exposure stage and the blooming stage, and inflorescences can be harvested from the exposure stage to the blooming stage. Furthermore, some aroma-related terpenoids were produced only in specific parts of *O. longilobus* plants and at specific inflorescence developmental stages.

The results indicated that the relative terpenoid content was higher than that of the other metabolites in O. longilobus leaves and inflorescences (**Supplementary File 4**).Specifically, the terpenoid relative contents in the leaves and inflorescences at the exposure, initial opening, and blooming stages were 48.57%, 42.27%, 41.41%, and 46.75%, respectively. The compounds with relatively high contents in the leaves were identified as linalyl acetate; epizonarene; bicyclo[2.2.1]heptane-2,5-dione; 1,7,7-trimethyl-,7-oxabicyclo[4.1.0]heptan-2-one, 3-methyl-6-(1-methylethyl)-; and 2,6-octadien-1-ol, 3,7-dimethyl-. The compounds with relatively high contents in the inflorescences at the exposure stage were linalyl acetate; thujone; endo-borneol; fenchol; and eucalyptol. The compounds with relatively high contents in the inflorescences at the initial opening stage were endo-borneol;.beta.-phellandrene; eucalyptol; alpha-curcumene; and (+)-alpha-pinene. The compounds with relatively high contents in the inflorescences at the blooming stage were linalyl acetate; bicyclo[3.1.0]hexan-3-one, 4-methyl-1-(1-methylethyl)-; alpha-curcumene; thujone; and Sabenene. The relative contents of linalyl acetate, thujone, endo-borneol, eucalyptol, and alpha-curcumene were higher than those of other terpenoids in O. longilobus leaves and inflorescences. Linalyl acetate peaked in leaves and inflorescences at the exposure and blooming stages. Thujone peaked in the inflorescences at the exposure and blooming stages. Endo-borneol and eucalyptol peaked in the inflorescences at the exposure and initial opening stages. Alpha-curcumene peaked in the inflorescences at the initial opening and blooming stages. These terpenoids may be important sources of the floral scent in *O. longilobus* leaves and inflorescences.

### Analysis of DAMs

The comparison between the leaves and the inflorescences at the initial opening stage resulted in the most DAMs (i.e., 1,078), of which 739 were up-regulated and 339 were down-regulated (**Table 1**; CTLeaf-vs-CTFlower). The comparison between the inflorescences at the exposure stage and those at the initial opening stage resulted in the fewest DAMs (i.e., 342), of which 30 were up-regulated and 312 were down-regulated (**Table 1**; CTcolour-vs-CTFlower). The results for the other comparisons are provided in **Table 1**. These DAMs were mainly related to terpenoids, esters, and heterocyclic compounds, reflecting the substantial differences in the terpenoids between inflorescences and between the leaves and inflorescences at different developmental stages.

**Table 1:** Comparison of the DAMs among *Opisthopappus longilobus* leaves and flowers.

Group name	All sig diff	Down regulated	Up regulated
CTLeaf_vs_CTcolour	941	177	764
CTLeaf_vs_CTFlower	1078	339	739
CTLeaf_vs_CTBloom	818	97	721
CTcolour_vs_CTFlower	342	312	30
CTcolour_vs_CTBloom	525	159	366
CTFlower_vs_CTBloom	776	251	525

Group name: names of the groups in each comparison; All sig diff: number of significantly different DAMs; Down-regulated: number of down-regulated DAMs; Up-regulated: number of up-regulated DAMs; CTLeaf: leaves of O. *longilobus*; CTcolour: inflorescences at the exposure stage of *O. longilobus*; CTFlower: inflorescences at the initial opening stage of *O. longilobus*; CTBloom: inflorescences at the blooming stage of *O. longilobus*.

### Analysis of differentially accumulated terpenoids

Of the DAMs in the *O. longilobus* leaves and inflorescences, the most diverse were terpenoids, esters, and heterocyclic compounds. Among these compounds, the terpenoids accounted for the largest proportion of DAMs in *O. longilobus*. Thus, we analyzed their contents more precisely. A total of 72, 176, and 125 differentially accumulated terpenoids were detected by comparing inflorescences at the exposure and initial opening stages, inflorescences at the initial opening and blooming stages, and inflorescences at the exposure and blooming stages, respectively. The contents of the differentially accumulated terpenoids initially increased and then decreased during the inflorescence development stages (i.e., exposure stage to the initial opening stage and then to the blooming stage). The contents of some of the terpenoids then gradually increased or decreased. The six compounds with increasing contents were petasitene; bicyclo[5.2.0]nonane, 2-methylene-4,8,8-trimethyl-4-vinyl-; [1aR-(1a.alpha.,4.alpha.,4a.beta.,7b.alpha.)]-1a,2,3,4,4a,5,6,7b-octahydro-1,1,4,7-tetramethyl-1H-cycloprop[e]azulene; 2-cyclohexen-1-one, 2-hydroxy-3-methyl-6-(1-methylethyl)-; neric acid; and cyclohexanol, 3-ethenyl-3-methyl-2-(1-methylethenyl)-6-(1-methylethyl)-, [1R-(1.alpha.,2.alpha.,3.beta.,6.alpha.)]-. The compound with decreasing contents was identified as 7-octen-2-ol, 2,6-dimethyl-. We detected 252, 215, and 195 terpenoids differentially accumulated in leaves and inflorescences at initial opening, in leaves and inflorescences at the exposure stage, and in leaves and inflorescences at the blooming stage, respectively. As the flowers bloomed, the number of differentially accumulated terpenoids between the leaves and the inflorescences gradually decreased.

### Illumina sequencing and assembly results

To explore the genes involved in regulating the synthesis of terpenoids in *O. longilobus*, we sequenced the transcriptomes of the leaves, buds, and inflorescences at the exposure, initial opening, and blooming stages using Illumina sequencing technology. The three replicates of leaves at the flowering stage produced 22,918,336, 22,998,276, and 23,317,148 high-quality clean reads. The three replicates of buds produced 38,453,876, 22,519,010, and 21,108,076 high-quality clean reads. The three replicates of inflorescences at the exposure stage produced 26,119,773, 25,972,917, and 22,125,584 high-quality clean reads. The three replicates of inflorescences at the initial opening stage produced 22,396,054, 27,633,846, and 23,343,734 high-quality clean reads. The three replicates of inflorescences at the blooming stage produced 23,498,228, 26,952,801, and 26,823,554 high-quality clean reads. These high-quality reads were subsequently assembled to generate 95,114 unigene sequences, with a total size of 76,120,922 bp and an N50 of 1,280 bp. The maximum and minimum lengths were 14,400 and 201 bp, respectively. The average length was 800 bp. The overall assembly quality was good (**Table 2**).

**Table 2:** *De novo* assembly results.

Genes Num	N50	Max length	Min length	Average length	Total assembled bases
95114	1280	14400	201	800	76120922

### Functional annotation and classification of genes

**Figure 3:**
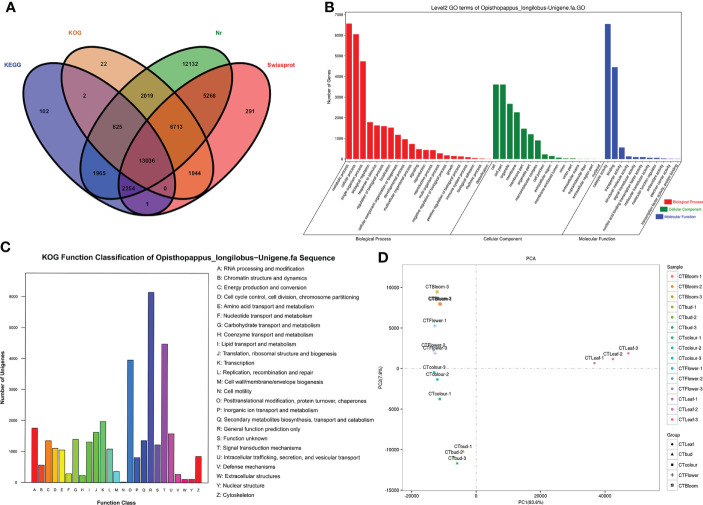
*Opisthopappus longilobus* transcriptome analysis results **(A)** Venn diagram of unigenes functionally annotated according to four major databases. **(B)** Histograms of the GO classifications. The unigenes were divided into the three main GO categories (biological process, cellular component, and molecular function). The x-axis lists the GO terms in the three categories, whereas the y-axis presents the number of unigenes. **(C)** KOG classification map. A total of 35,012 sequences with KOG classifications in 25 categories are presented. **(D)** Results of the principal component analysis of the *O. longilobus* transcriptome.

The 95,114 unigenes were functionally annotated on the basis of four major databases. A total of 45,674 unigenes were annotated. Specifically, 44,212 unigenes were annotated according to the Nr database, 28,607 unigenes were annotated using the Swiss-Prot database, 23,661 unigenes were annotated on the basis of the KOG database, and 18,185 unigenes were annotated according to the KEGG database (**Figure 3A**). However, 49,440 unigenes were not functionally annotated.

On the basis of the Nr-based functional annotation, we assigned GO terms to the unigenes. A total of 63,706 unigenes were divided into 49 functional categories, including 31,706 unigenes annotated with 22 GO terms from the biological process category, 18,568 unigenes annotated with 16 GO terms from the cellular component category, and 13,432 unigenes annotated with 11 GO terms from the molecular function category (**Figure 3B**). The common GO terms in the biological process category were ‘metabolic process,’ ‘cellular process,’ and ‘single-organism process,’ which were assigned to 7,402, 6,658, and 5,180 unigenes, respectively. In the cellular component category, the most enriched GO terms were ‘cell,’ ‘cell part,’ and ‘organelle,’ which were assigned to 4,260, 4,260, and 3,004 unigenes, respectively. In the molecular function category, the most common GO terms were ‘catalytic activity’ and ‘binding,’ which were assigned to 7,208 and 5,055 unigenes, respectively. Additionally, of the assigned GO terms in the three main GO categories, ‘locomotion,’ ‘extracellular region part,’ and ‘nucleoid’ were the least common (i.e., only two unigenes each).

The unigenes were also functionally annotated according to the KOG classifications (**Figure 3C**). Among the 25 functional categories in the KOG database, ‘General function prediction only’ (6,147, 17.6%), ‘Signal transduction mechanisms’ (4,478, 12.8%), and ‘Posttranslational modification, protein turnover, chaperones’ (3,958, 11.3%) had the most unigenes. In contrast, ‘Nuclear structure’ (115, 0.3%), ‘Extracellular structures’ (111, 0.3%), and ‘Cell motility’ (19, 0.05%) had the fewest unigenes.

The functional characterization on the basis of the enriched KEGG pathways among the unigenes was useful for clarifying the metabolic pathways associated with the complex biological activities in *O. longilobus*. A total of 9,346 unigenes were assigned to a KEGG pathway (**Table 3**). The most enriched KEGG pathways were ‘Metabolic pathways’ and ‘Biosynthesis of secondary metabolites,’ which were assigned to 3,695 (39.54%) and 1,986 (21.25%) unigenes, respectively, indicating that a substantial abundance of secondary metabolites was synthesized during the *O. longilobus* leaf, bud, and inflorescence developmental stages because the related genes were highly expressed.

**Table 3:** Enriched KEGG pathways among the *Opisthopappus longilobus* unigenes.

#	Pathway	All unigenes with pathway annotation (9346)	Pathway ID
1	Metabolic Pathways	3695 (39.54%)	ko01100
2	Biosynthesis of secondary metabolites	1986 (21.25%)	ko01110
3	Carbon metabolism	527 (5.64%)	ko01200
4	Protein processing in endoplasmic reticulum	492 (5.26%)	ko04141
5	Spliceosome	467 (5%)	ko03040
6	Plant hormone signal transduction	465 (4.98%)	ko04075
7	Plant-pathogen interaction	465 (4.98%)	ko04626
8	Ribosome	454 (4.86%)	ko03010
9	Biosynthesis of amino acids	430 (4.6%)	ko01230
10	Endocytosis	412 (4.41%)	ko04144
11	RNA transport	351 (3.76%)	ko03013
12	Purine metabolism	302 (3.23%)	ko00230
13	Ubiquitin mediated proteolysis	301 (3.22%)	ko04120
14	Oxidative phosphorylation	267 (2.86%)	ko00190
15	Starch and sucrose metabolism	262 (2.8%)	ko00500
16	Amonoacyl-tRNA biosynthesis	259 (2.77%)	ko00970
17	Pyramidine metabolism	256 (2.74%)	ko00240
18	Phenylpropanoid biosynthesis	252 (2.7%)	ko00940
19	Glycolysis/Gluconeogenesis	247 (2.64%)	ko00010
20	mRNA surveillance pathway	237 (2.54%)	ko03015
21	MAPK signalling pathway plant	234 (2.5%)	ko04016
22	RNA degradation	233 (2.49%)	ko03018
23	Nucleotide excision repair	218 (2.33%)	ko03420
24	Pyruvate metabolism	209 (2.24%)	ko00620
25	Amino sugar and nucleotide sugar metabolism	203 (2.17%)	ko00520
26	Homologous recombination	201 (2.15%)	ko03440
27	Fatty acid metabolism	200 (2.14%)	ko01212
28	DNA replication	195 (2.09%)	ko03030
29	Glycerophospholipid metabolism	191 (2.04%)	ko00564
30	Mismatch repair	179 (1.92%)	ko03430

### Principal component analysis of the transcriptome

We performed a principal component analysis of the *O. longilobus* transcriptome to elucidate the differences between the samples in each group and the degree of variation between samples within the group as a whole. For the *O. longilobus* leaf, bud, and inflorescence data, PC1 and PC2 accounted for 83.6% and 7.8% of the total variance, respectively (**Figure 3D**). There was a large overall metabolic difference between the samples, but a relatively small variation within the group. Moreover, there was a greater separation between the leaves and inflorescences than between the inflorescences at different developmental stages.

### Analysis of the DEGs in the leaves, buds, and inflorescences of *Opisthopappus longilobus*

**Figure 4:**
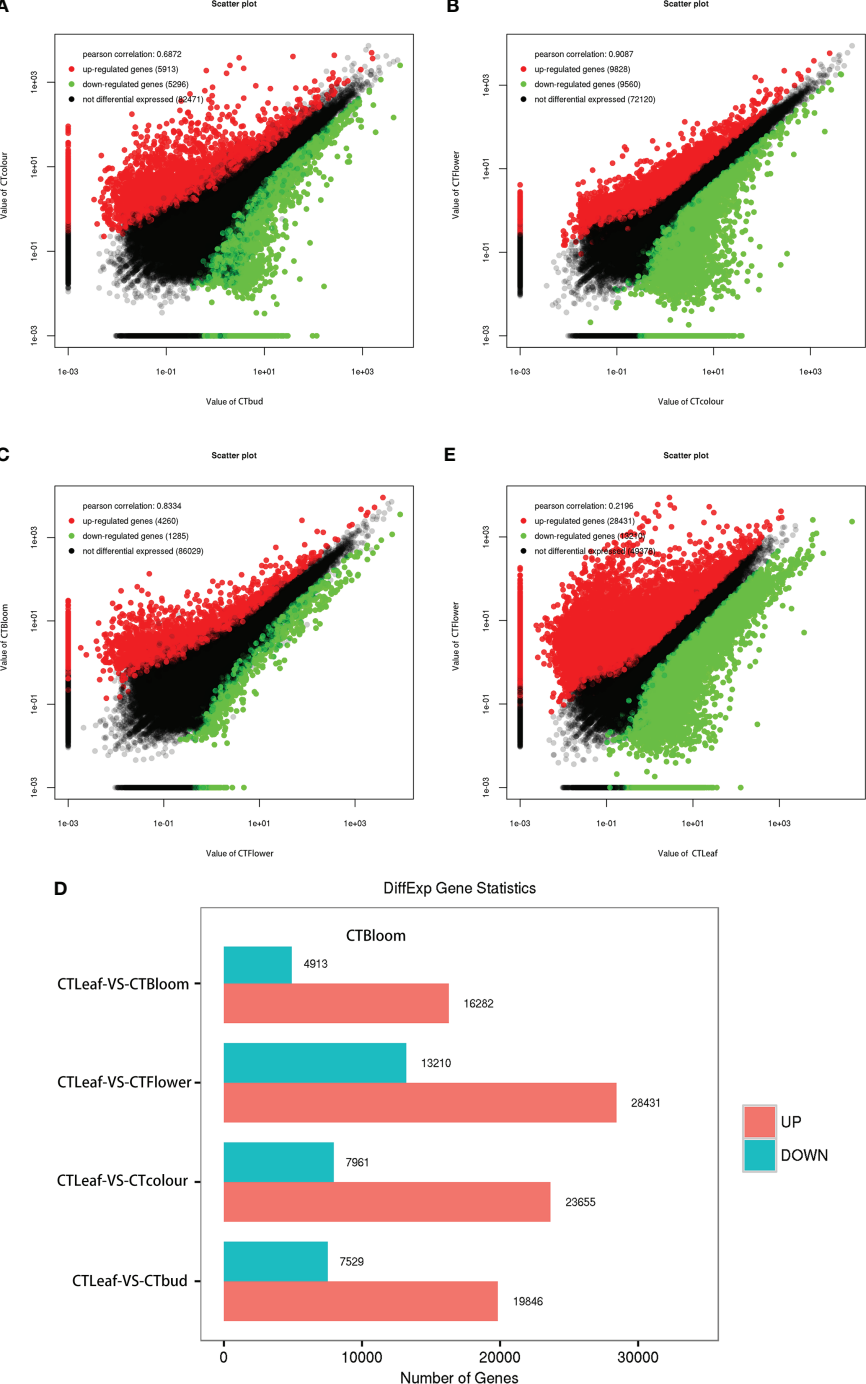
Differentially expressed genes in the leaves, buds, and inflorescences of *O. longilobus*. **(A)** Scatter plot of the up-regulated and down-regulated genes detected by the comparison between the buds and inflorescences at the exposure stage. **(B)** Scatter plot of the up-regulated and down-regulated genes detected by the comparison between the inflorescences at the exposure and initial opening stages. **(C)** Scatter plot of the up-regulated and down-regulated genes detected by the comparison between the inflorescences at the initial opening and blooming stages. **(D)** Changes in the expression of the DEGs between the leaves and the buds as well as the inflorescences at the exposure, initial opening, and blooming stages. **(E)** Scatter plot of the up-regulated and down-regulated genes detected by the comparison between the leaves and the inflorescences at the initial opening stage.

The analysis of all *O. longilobus* samples detected 94,400 DEGs, accounting for 99.25% of the total number of reference genes. Additionally, the gene expression levels were very high. The DEGs between the buds and inflorescences at two consecutive stages were identified using the following thresholds: FDR < 0.05 and |log_2_(fold-change)| > 1. Accordingly, 11,209 DEGs were identified between the buds and the inflorescences at the exposure stage. Compared with the buds, 5,913 and 5,296 DEGs had significantly up-regulated and down-regulated expression levels in the inflorescences at the exposure stage, respectively (**Figure 4A**; **Supplementary File 5**). A total of 19,388 DEGs were identified between the inflorescences at the exposure and initial opening stages. Compared with the inflorescences at the exposure stage, 9,828 and 9,560 DEGs were expressed at significantly higher and lower levels in the inflorescences at the initial opening stage, respectively (**Figure 4B**; **Supplementary File 6**). In contrast, 5,545 DEGs were identified between the inflorescences at the initial opening and blooming stages. Compared with the inflorescences at the initial opening stage, 4,260 and 1,285 DEGs had significantly up-regulated and down-regulated expression levels in the inflorescences at the blooming stage, respectively (**Figure 4C**; **Supplementary File 7**). Therefore, the clear difference in the number of DEGs detected in these two comparisons may reflect the changes in the contents of aromatic substances in developing buds and inflorescences.

We also screened the DEGs between the *O. longilobus* leaves and the buds as well as the inflorescences at the exposure, initial opening, and blooming stages. More DEGs were detected in the comparison between the leaves and the inflorescences at the initial opening stage than in the other comparisons (**Figure 4D**). A total of 41,641 DEGs were identified between the leaves and the inflorescences at the initial opening stage. Compared with the leaves, 28,431 and 13,210 DEGs were expressed at significantly higher and lower levels in the inflorescences at the initial opening stage, respectively (**Figure 4E**; **Supplementary File 8**). Therefore, we detected considerable differences in gene expression between the leaves and the buds as well as the inflorescences, especially the inflorescences at the initial opening stage. Next, we analyzed the expression of genes related to terpene synthesis in *O. longilobus* leaves, buds, and inflorescences.

### Analysis of the expression of genes related to terpene biosynthesis

**Figure 5:**
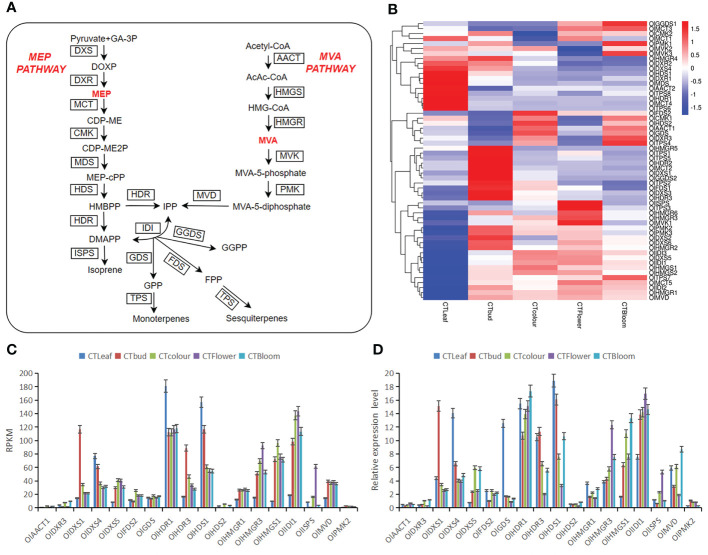
Important factors mediating the terpene biosynthesis pathway in *O. longilobus* and the expression patterns of 18 selected genes determined by the qRT-PCR analysis. **(A)** Important factors in the *O. longilobus* terpene biosynthesis pathway. GA-3P, glyceraldehyde-3-phosphate; DOXP, 1-deoxy-D-xylulose 5-phosphate; DXS, DOXP synthase; DXR, DOXP reductoisomerase; MEP, 2-C-methyl-D-erythritol 4-phosphate; MCT, 2-C-methyl-D-erythritol 4-phosphate cytidylyltransferase; CDP-ME, 4-(cytidine 5′-diphospho)-2-C-methyl-D-erythritol; OlK, CDP-ME kinase; CDP-ME2P, 4-(cytidine 5′-diphospho)-2-C-methyl-D-erythritol phosphate; MDS, 2-C-methyl-D-erythritol 2,4-cyclodiphosphate synthase; MEP-cPP, 2-C-methyl-D-erythritol 2,4-cyclodiphosphate; HDS, (E)-4-hydroxy-3-methylbut-2-enyl diphosphate synthase; HMBPP, (E)-4-hydroxy-3-methylbut-2-enyl diphosphate; HDR, (E)-4-hydroxy-3-methylbut-2-enyl diphosphate reductase; DMAPP, dimethylallyl pyrophosphate; ISPS, isoprene synthase; IDI, isopentenyl diphosphate isomerase; IPP, isopentenyl pyrophosphate; GDS, geranyl diphosphate synthase; GPP, geranyl diphosphate; TPS, terpene synthase; FDS, farnesyl diphosphate synthase; FPP, farnesyl diphosphate; GGDS, geranyl geranyl diphosphate synthase; GGPP, geranyl geranyl diphosphate; AACT, acetoacetyl-CoA thiolase; AcAc-CoA, acetoacetyl-CoA; HMG-CoA, 3-hydroxy-3-methylglutaryl-CoA; HMGS, HMG-CoA synthase; HMGR, HMG-CoA reductase; MVA, mevalonate; MVK, mevalonate kinase; MVA-5-phosphate, mevalonate-5-phosphate; PMK, phosphomevalonate kinase; MVA-5-diphosphate, mevalonate-5-diphosphate; MVD, mevalonate diphosphate decarboxylase. The names of enzymes are in boxes. **(B)** Heat map of the gene expression associated with terpene synthesis in *O. longilobus* leaves, buds, and inflorescences. The columns and rows in the heat map represent the samples and genes, respectively. The color scale indicates the fold-change in gene expression, with red and blue reflecting high and low expression levels, respectively. **(C)** Expression levels of 18 genes presented in terms of the reads per kilobase per million reads (RPKM) value. Error bars represent the standard deviation from three replicates. **(D)** Relative expression levels of 18 genes determined by the qRT-PCR analysis. Error bars represent the standard deviation from three replicates.

To explore the differences in the contents of aromatic substances between *O. longilobus* leaves and buds as well as inflorescences, we determined the differences in the expression of *O. longilobus* terpene biosynthesis pathway genes important for floral aroma formation (**Figure 5A**). The active expression of these genes resulted in the synthesis and accumulation of 308 diverse terpenoids in the *O. longilobus* leaves, buds, and inflorescences at the exposure, initial opening, and blooming stages. According to the terpene metabolic pathway (Feng et al., 2014), 56 genes related to terpene synthesis were identified (**Figure 5B**; **Supplementary File 9**). These genes, which may be important for molecular biology research and the breeding of *O. longilobus*, encoded the following proteins: acetoacetyl-CoA thiolase (AACT), 3-hydroxy-3-methylglutaryl-CoA synthase (HMGS), 3-hydroxy-3-methylglutaryl CoA reductase (HMGR), mevalonate kinase (MVK), phosphomevalonate kinase (PMK), mevalonate diphosphate decarboxylase (MVD), 1-deoxy-D-xylulose 5-phosphate synthase (DXS), 1-deoxy-D-xylulose 5-phosphate reductoisomerase (DXR), 2-C-methyl-D-erythritol 4-phosphate cytidylyltransferase (MCT), 4-(cytidine 5′-diphospho)-2-C-methyl-D-erythritol kinase (CMK), 2-C-methyl-D-erythritol-2,4-cyclodiphosphate synthase (MDS), (E)-4-hydroxy-3-methylbut-2-enyl diphosphate synthase (HDS), (E)-4-hydroxy-3-methylbut-2-enyl diphosphate reductase (HDR), isopentenyl diphosphate isomerase (IDI), farnesyl diphosphate synthase (FDS), geranyl diphosphate synthase (GDS), geranyl diphosphate synthase (GGDS), terpene synthase (TPS), and isoprene synthase (ISPS).

We identified 31 terpene synthesis-related DEGs between the *O. longilobus* leaves and inflorescences at the exposure stage. Compared with the leaves, the expression levels of 20 and 11 genes were up-regulated (e.g., *OlDXS1*, *OlDXS5*, and *OlFDS2*) and down-regulated (e.g., *OlDXR1*, *OlDXS4*, and *OlHDR1*) in the inflorescences at the exposure stage, respectively. We identified 34 terpene synthesis-related DEGs between the leaves and inflorescences at the initial opening stage. Compared with the leaves, the expression levels of 18 and 16 genes were up-regulated (e.g., *OlDXS5*, *OlHMGR1*, and *OlHMGR2*) and down-regulated (e.g., *OlCMK1*, *ODXR1*, and *OlDXR3*) in the inflorescences at the initial opening stage, respectively. We identified 24 terpene synthesis-related DEGs between the leaves and inflorescences at the blooming stage. Compared with the leaves, the expression levels of 13 and 11 genes were up-regulated (e.g., *OlPMK2*, *OlTPS1*, and *OlTPS3*) and down-regulated (e.g., *OlISPS*, *OlMCT4*, and *OlMDS*) in the inflorescences at the blooming stage. The significant differences in gene expression between the *O. longilobus* leaves and inflorescences are likely the molecular basis of the differences in the terpenoid production and accumulation between these tissues. Compared with the *O. longilobus* leaves, the expression levels of *OlDXS5*, *OlHMGR1*, *OlHMGR3*, *OlHMGS1*, *OlHMGS2*, *OlIDI1*, *OlIDI3*, *OlMVD*, *OlMVK1*, and *OlTPS3* were up-regulated in the inflorescences at different developmental stages, whereas the expression levels of *OlMVK3*, *OlPMK2*, *OlMDS*, *OlDXR1*, *OlDXS4*, *OlHDR1*, *OlHDS1*, *OlHMGR4*, *OlHMGR5*, *OlISPS*, *OlMCT4*, *OlTPS1*, and *OlTPS6* were down-regulated. In a previous study, genes with an expression value less than 0.3 were designated as unexpressed genes (Kumar et al., 2016). On the basis of this threshold, *OlMVK1*, *OlPMK2*, *OlTPS1*, and *OlTPS3* were expressed in the inflorescences, but not in the leaves. This may have resulted in the accumulation of 11 terpenoids only in the inflorescences (e.g., 2-cyclohexen-1-one, 2-hydroxy-3-methyl-6-(1-methylethyl)-; bicyclo[3.1.1]heptan-3-ol, 6,6-dimethyl-2-methylene-; and squamulosone).

We identified 12 terpenoid synthesis-related DEGs between the inflorescences at the exposure and initial opening stages. Compared with the inflorescences at the exposure stage, the expression levels of five genes (*OlHMGR5*, *OlTPS1*, *OlTPS3*, *OlTPS5*, and *OlTPS6*) and seven genes (*OlCMK1*, *OlDXR3*, *OlGGDS2*, *OlHDS2*, *OlISPS*, *OlTPS4*, and *OlTPS8*) were up-regulated and down-regulated, respectively, in the inflorescences at the initial opening stage. We identified six terpenoid synthesis-related DEGs between the inflorescences at the initial opening and blooming stages. Compared with the inflorescences at the initial opening stage, the expression levels of five genes (*OlCMK1*, *OlDXR3*, *OlHDS2*, *OlISPS*, and *OlTPS4*) and one gene (*OlTPS5*) were up-regulated and down-regulated, respectively, in the inflorescences at the blooming stage. We identified two terpenoid synthesis-related DEGs between the inflorescences at the exposure and blooming stages. Compared with the inflorescences at the exposure stage, the expression levels of two genes (*OlHMGR4* and *OlGGDS2*) were down-regulated in the inflorescences at the blooming stage. The number of DEGs related to terpenoid synthesis gradually decreased as *O. longilobus* flowers developed (i.e., from the exposure to initial opening stages and then to the blooming stage). Some genes were up-regulated, whereas others were down-regulated. The genes related to terpenoid synthesis were most actively expressed in the inflorescences at the initial opening stage, which may be associated with the gradual increase in the contents of certain terpene metabolites (e.g., petasitene; bicyclo[5.2.0]nonane, 2-methylene-4,8,8-trimethyl-4-vinyl-; and [1aR-(1a.alpha.,4.alpha.,4a.beta.,7b.alpha.)]-1a,2,3,4,4a,5,6,7b-octahydro-1,1,4,7-tetramethyl-1H-cycloprop[e]azulene). In contrast, the contents of other terpene metabolites gradually decreased (e.g., 7-octen-2-ol, 2,6-dimethyl-).

### Gene expression analysis by qRT-PCR

To verify the RNA-seq data for the expression of specific genes in *O. longilobus* leaves and inflorescences, we random selected 18 genes for a qRT-PCR analysis. These genes are important for terpene biosynthesis in *O. longilobus*. The generated qRT-PCR data for all 18 genes were in accordance with the RNA-seq data (**Figures 5C, D**). These results indicated the transcriptome sequencing data were highly reliable and useful for further research on the *O. longilobus* genes involved in terpene biosynthesis.

### Integrated analysis of the transcriptome and metabolome data

**Figure 6:**
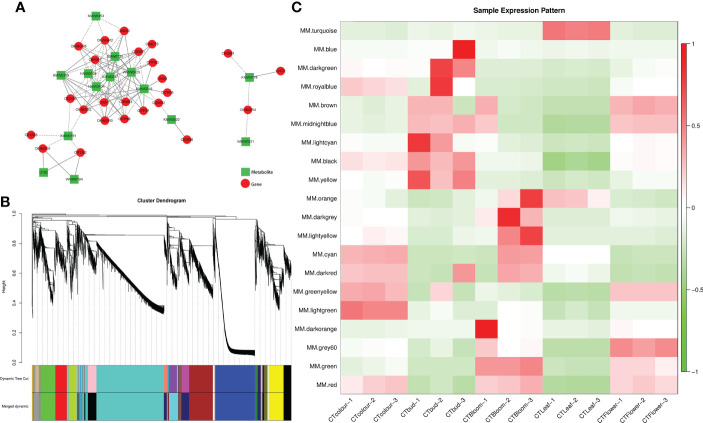
Combined analysis of the transcriptome and metabolome and weighted gene co-expression network analysis. **(A)** Correlation network according to gene expression and metabolite abundance. In the network, genes and metabolites are represented by red circles and green rectangles, respectively. Positive and negative correlations are indicated by solid and dashed lines, respectively. **(B)** Module-level clustering diagram. Dynamic Tree Cut divides the module according to the clustering result. Merged Dynamic divides the module according to the similarity in expression patterns. The subsequent analysis was performed using the merged module. In the tree graph, the longitudinal distance represents the distance between two nodes (i.e., genes), whereas the transverse distance is meaningless. **(C)** Heat map of the sample expression patterns. The x-axis presents the samples, whereas the y-axis presents the modules. The module eigenvalue was used to draw the graph. Red and green represent high and low expression levels, respectively. The figure reflects the expression pattern of each module in each sample.

To more thoroughly characterize the association between terpenoid production and the expression of the related genes in *O. longilobus*, we performed a correlation analysis using the transcriptome and metabolome data. A correlation network comprising 24 terpene synthesis-related genes and the top 14 terpene metabolites in terms of the relative contents in the leaves and inflorescences at different developmental stages was plotted (**Figure 6A**). A total of 97 pairs (i.e., correlations) were identified. There were many DEGs involved in the terpene synthesis pathway. Furthermore, there were many positive correlations between the DEGs and differentially accumulated terpene metabolites. More specifically, there were 51 and 46 positive and negative correlations, respectively. The correlation analysis indicated that the terpene metabolites with the most positive correlations with the DEGs were.beta.-phellandrene (KMW0247) (11 pairs), (+)-alpha-pinene (WMW0023) (11 pairs), and eucalyptol (KMW0218) (10 pairs). The terpene metabolites with the most negative correlations with the DEGs were bicyclo[2.2.1]heptane-2,5-dione, 1,7,7-trimethyl- (NMW0108) (eight pairs), 2,6-octadien-1-ol, 3,7-dimethyl- (NMW0104) (eight pairs), epizonarene (XMW0151) (five pairs), and 7-oxabicyclo[4.1.0]heptan-2-one, 3-methyl-6-(1-methylethyl)- (XMW0733) (five pairs).

### Weighted gene co-expression network analysis

A weighted gene co-expression network analysis is useful for examining the expression patterns of genes in multiple samples. It can elucidate the association between modules and specific traits or phenotypes by clustering genes with similar expression patterns (Zhang and Horvath, 2005), making it relevant for analyzing the expression patterns of genes related to terpene synthesis in *O. longilobus* leaves and inflorescences. A total of 23,465 genes were divided into 20 modules (**Figure 6B**). The turquoise module had the most genes (6,887 unigenes), followed by the blue (3,644 unigenes) and brown (2,399 unigenes) modules. The dark orange module had the fewest genes (66 unigenes). Furthermore, 33 of the 56 genes related to terpene synthesis were divided into eight modules, of which the turquoise module included 15 genes, whereas the yellow and cyan modules had four genes each. The red and blue modules included three genes each, the black module had one gene, as did the light cyan and brown modules.

The module eigenvalue reflects the comprehensive expression of all genes in the module in each sample. Thus, it was used to draw the heat map for the sample expression patterns. The genes in the turquoise module were highly expressed in the *O. longilobus* leaves but were expressed at relatively low levels in the inflorescences at different developmental stages (**Figure 6C**). The turquoise module had the most terpenoid synthesis-related genes. These results may help to explain why the highest relative content for all terpenoid metabolites was detected in the leaves (48.57%). The genes in the yellow, blue, and light cyan modules were highly expressed in the buds but were expressed at low levels in the leaves and the inflorescences at the exposure, initial opening, and blooming stages. The genes in the red module were highly expressed in the inflorescences at the exposure, initial opening, and blooming stages, but they were expressed at low levels in the leaves and buds. The genes in the cyan module were highly expressed in the inflorescences at the exposure and blooming stages, which was in contrast to their low expression levels in the leaves, buds, and inflorescences at the initial opening stage. The genes in the black module were highly expressed in the buds and inflorescences at the exposure and initial opening stages, whereas they were expressed at low levels in the leaves and inflorescences at the blooming stage. The genes in the brown module were highly expressed in the buds and inflorescences at the initial opening stage, whereas their expression levels were low in the leaves and inflorescences at the exposure and blooming stages.

**Figure 7:**
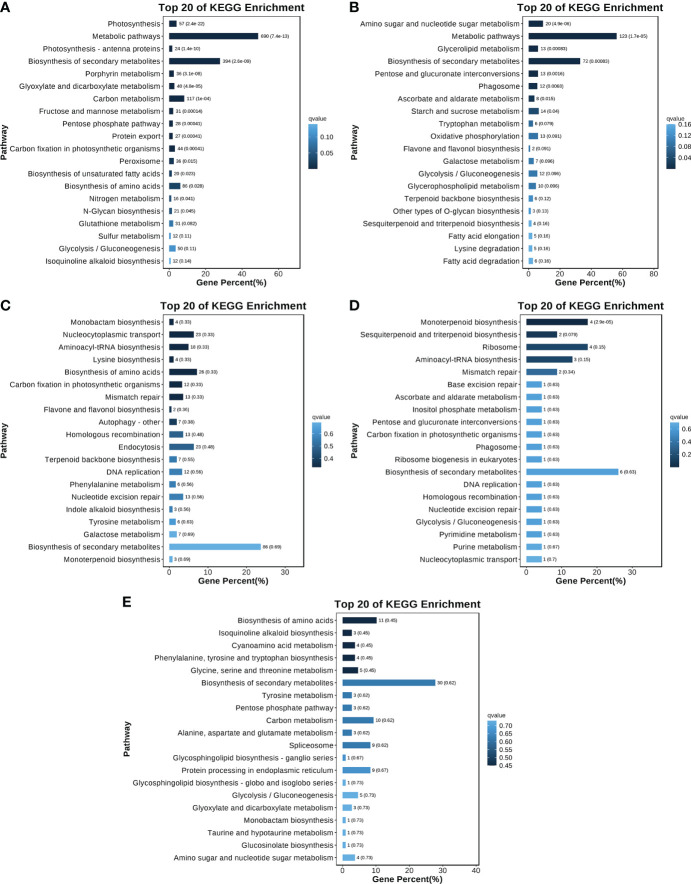
KEGG analysis of different modules. **(A–E)** Twenty most enriched KEGG pathways in the turquoise, red, blue, light cyan, and cyan modules, respectively. The y-axis presents the KEGG pathways, whereas the x-axis presents the proportion of the genes assigned to a particular pathway. Increases in the color intensity reflect decreases in the P/Q value. The values next to each bar represent the gene number and the P/Q value.

Because of the significant changes in gene expression patterns and the identification of genes related to terpenoid synthesis, the turquoise, red, blue, light cyan, and cyan modules were selected for a KEGG analysis (**Figures 7A–E**). The most enriched KEGG pathways among the genes in the turquoise and red modules were ‘Metabolic pathways’ (690 and 123 genes in the turquoise and red modules, respectively) and ‘Biosynthesis of secondary metabolites’ (394 and 72 in the turquoise and red modules, respectively). ‘Biosynthesis of secondary metabolites’ was the most enriched KEGG pathway in the blue (86 genes), cyan (30 genes), and light cyan (six genes) modules. These findings indicated that the genes related to secondary metabolic pathways (e.g., terpenoid synthesis) were actively and differentially expressed in each group of *O. longilobus* samples. These results were consistent with the results of the DEG analysis, indicating that the leaves and inflorescences at different developmental stages accumulated various aromatic metabolites.

### Analysis of the composition of the essential oil extracted from the leaves

Essential oil was extracted from *O. longilobus* leaves *via* steam distillation (extraction rate of 2.07‰). The essential oil was black, and the subsequent analysis of its composition detected 380 metabolites (**Supplementary File 10**). We classified and analyzed the relative contents of these metabolites. Terpenoids were the most common metabolites (87), followed by esters (76), heterocyclic compounds (58), alcohols (24), ketones (24), aromatics (23), hydrocarbons (17), amines (17), aldehydes (16), halogenated hydrocarbons (13), and nitrogen compounds (10). The less common metabolites were phenols (6), acids (5), sulfur compounds (3), and compounds from other classes (1). The compounds with the three highest relative contents were terpenoids, including [1S-(1.alpha.,4.beta.,5.alpha.)]-4-methyl-1-(1-methylethyl)-bicyclo[3.1.0]hexan-3-one (23.69%), (+)-2-bornanone (11.11%), and cyclohexene, 4-ethenyl-4-methyl-3-(1-methylethenyl)-1-(1-methylethyl)-, (3R-trans)- (5.98%). The most abundant aromatic substances were terpenoids, accounting for up to 73.79% of the aromatic substances, which was consistent with the above-mentioned results of the analyses of the leaves and inflorescences, indicating that these abundant terpenoids contribute to the special *O. longilobus* aroma. Therefore, *O. longilobus* leaves may be a good source of essential oil, which can be collected in three seasons (i.e., spring, summer, and autumn), possibly leading to relatively high yields. These results lay the foundation for future attempts to further develop *O. longilobus* resources and increase the economic value of this plant species.

## Discussion

### Many significant differentially expressed genes encoding enzymes may be useful candidate genes mediating terpenoid synthesis in *Opisthopappus longilobus*

The regulation of terpenoid synthesis by various pathways in flowers has been relatively well characterized. Although there have been considerable advances in the research on the biochemical pathways and molecular regulatory mechanisms associated with floral volatiles, molecular studies on *O. longilobus* floral volatiles are limited and there is relatively little available transcriptome and metabolome information. Therefore, we thoroughly explored the components and gene regulatory pathways of aromatic substances in *O. longilobus* leaves and inflorescences on the basis of earlier studies on other crops.

In a recent study, He et al. analyzed the differences in the expression of genes related to aroma synthesis in Chinese narcissus; they identified 46 significant DEGs encoding enzymes related to the biosynthesis of floral volatiles (He et al., 2020). These findings are important for further clarifying the mechanism underlying volatile biosynthesis in daffodils. The MVA and MEP pathways are two different metabolic pathways for terpene biosynthesis (Nagegowda, 2010; Zhong et al., 2022). Both pathways are active in *O. longilobus* leaves, buds, and inflorescences at different developmental stages. By examining the expression of genes involved in these two pathways influencing terpenoid accumulation, we detected 56 significant DEGs encoding enzymes related to terpenoid synthesis in *O. longilobus* (e.g., *OlHMGR1*, *OlDXS5*, *OlPMK2*, *OlMVK1*, and *OlTPS1*). These may be useful candidate genes for future molecular biological research and the breeding of *O. longilobus*. The active expression of these genes may have increased the diversity of the terpenoids (308) in the leaves, buds, and inflorescences at the exposure, initial opening, and blooming stages.

In a recent study, HS-SPME was combined with GC-MS to identify volatile compounds in the leaves of *O. longilobus* and four other chrysanthemum species, which revealed that most of the detected compounds were terpenes. Moreover, many compounds were released only by the *O. longilobus* flowers (Guo et al., 2020). For example, 1,8-cineole was detected at relatively high levels in *O. longilobus*, whereas it was undetectable or detected at low levels in the other species, suggesting it may be responsible for the formation of the distinct *O. longilobus* aroma (Guo et al., 2020). Through a correlation analysis, Guo et al. determined that the volatilization of terpenoids was highly correlated with the density of capitate trichomes; these structures had the highest terpenoid content and density in *O. longilobus* (Guo et al., 2020). Zhang et al. studied terpenoid diversity and biosynthesis in the flowers of 44 chrysanthemum species/varieties; their principal component analysis indicated that six monoterpenoids and five sesquiterpenoids were the main terpenoids that accumulated internally before being released (Zhang et al., 2021). In our study, as the *O. longilobus* flowers opened, the number of DEGs related to the differential terpene synthesis between the leaves and inflorescences first increased and then decreased, with more up-regulated genes than down-regulated genes (e.g., *OlHMGR1*, *OlDXS5*, *OlTPS4*, and *OlTPS6*). In contrast, the number of differentially accumulated terpene metabolites decreased gradually. This may be related to the accumulation of various terpenoids in the leaves (Guo et al., 2020). Moreover, *OlPMK2*, *OlMVK1*, *OlTPS1*, and *OlTPS3* expression was detected in the inflorescences at the exposure, initial opening, and blooming stages, but not in the leaves. The exclusive expression of these genes in the inflorescences may lead to the accumulation of 11 different terpenoids specifically in the inflorescences (e.g., squamulosone; p-mentha-1,5,8-triene; and 6,7-dimethyl-1,2,3,5,8,8a-hexahydronaphthalene).

We determined that *O. longilobus* leaves and inflorescences differ significantly regarding gene expression, which may be the molecular basis for the differences in the types of terpenoids in these plant parts. There were more DEGs revealed in the comparison between inflorescences at the exposure and initial opening stages than in the other comparisons of inflorescence stages. The differences in gene expression in *O. longilobus* were generally consistent with the differences in metabolites. There were many DEGs and DAMs between the leaves and the buds as well as the inflorescences at the exposure, initial opening, and blooming stages. The comparison between the leaves and inflorescences at the initial opening stage had the most DEGs and up-regulated DAMs, indicative of the considerable difference between metabolite synthesis and the expression of the related genes in the leaves and inflorescences. This may be related to the diversity in the types and contents of aromatic substances in these plant tissues. In the developing inflorescences, there were fewer DEGs and DAMs between the inflorescences at the initial opening and blooming stages than between the inflorescences at the exposure and initial opening stages. Hence, during the development of *O. longilobus* leaves, buds, and inflorescences, secondary metabolites, including aromatic substances, are actively being synthesized, thereby promoting aroma formation.

In an earlier study on the expression patterns of genes related to terpene biosynthesis in *Cinnamomum* species, three monoterpene synthase genes (*TPS14-like1*, *TPS14-like2*, and *TPS14-like3*) were more highly expressed in the cinnamomum-type plants than in the linalool-type plants; this finding was confirmed by a real-time PCR analysis (Chen et al., 2018). After studying six additional full-length candidate *TPS* genes in *Cinnamomum* species, Ma et al. showed that *CbTPS1*, *CbTPS2*, and *CbTPS3* encode enzymes that catalyze monoterpene formation, whereas the enzymes encoded by *CbTPS4*, *CbTPS5*, and *CbTPS6* catalyze monoterpene and sesquiterpene production. However, *CbTPS7* encodes a linalool/nerolol synthase (Ma et al., 2022). These *CbTPS* genes are responsible for the synthesis of 10 monoterpenes and 14 sesquiterpenes in *Cinnamomum* species, including monoterpenes and sesquiterpenes produced in leaves (Ma et al., 2022). This previous study provided researchers with important reference material for the synthesis of terpenoids in the *Cinnamomum* leaf essential oil. Another recent study identified the *Aquilaria TPS* gene family using bioinformatics methods and demonstrated that *AsTPS* gene expression might be regulated by stress and jasmonic acid. The transient expression of *AsERF1* in *Nicotiana benthamiana* significantly enhances the activation of the *AsTPS1* promoter (Li et al., 2021b). These results suggest that AsERF1 may influence sesquiterpene biosynthesis by regulating *AsTPS1* expression. By examining tissue-specific expression, Wang et al. revealed that *Cymbidium faberi TPS* expression levels are highest in the flowers, followed by the leaves and pseudobulbs. Additionally, *CfTPS12*, *CfTPS18*, *CfTPS23*, and *CfTPS28* are mainly expressed during the full flowering stage (Wang et al., 2021b). These findings are relevant for future investigations on the *TPS* genes in orchids.

The number of DEGs related to differential terpene synthesis decreased gradually as the *O. longilobus* inflorescences developed (from the exposure to the initial opening stages and then to the blooming stage). The expression levels of some genes were up-regulated, whereas the expression levels of other genes were down-regulated (e.g., *OlCMK1*, *OlTPS1*, and *OlTPS3*). This may help to explain the gradual increase in the contents of some terpenoids (e.g., petasitene) as well as the gradual decrease in the contents of other terpenoids (e.g., 7-octen-2-ol, 2,6-dimethyl-). There were no terpenoid-related genes specifically expressed in the inflorescences at the initial opening stage, which was consistent with the absence of specific terpenoids in the inflorescences at the initial opening stage. The expression levels of *OlHDS2*, *OlTPS8*, *OlCMK1*, and *OlMVK3*, which were specifically expressed in the inflorescences at the exposure and blooming stages, initially decreased and then increased. Both *OlPMK1* and *OlAACT2* were specifically expressed in the inflorescences at the blooming stage. The expression of *OlHDS2*, *OlTPS8*, *OlCMK1*, *OlMVK3*, *OlPMK1*, and *OlAACT2* may specifically induce the accumulation of four terpenoids in the inflorescences at the exposure and blooming stages, namely bicyclo[7.2.0]undec-4-ene, 4,11,11-trimethyl-8-methylene-, [1R-(1R*,4Z,9S*)]-; cyclohexanol, 1-methyl-4-(1-methylethyl)-, cis-; cyclopropanecarboxylic acid, 2,2-dimethyl-3-(2-methyl-1-propenyl)-, (1R-trans)-; and bornyl acetate.

Liu et al. analyzed the volatile organic compounds in *Albizia julibrissin* and determined that *AjTPS2*, *AjTPS5*, *AjTPS7*, *AjTPS9*, and *AjTPS10* are among 11 terpenoid synthetase genes involved in the synthesis of volatile oil terpenoids (Liu et al., 2021a). Terpenoid synthetase catalyzes the conversion of the corresponding precursor compounds into various terpenoids. Isoprene, which is the basic component of terpenoids, is an important raw material for the synthesis of rubber, pesticides, medicines, spices, and adhesives (Zhong et al., 2022). In the current study, we analyzed the gene regulatory network of terpenoids in *O. longilobus*. We also examined the candidate genes related to terpenoid synthesis. The data presented herein may provide the theoretical basis for future gene cloning and molecular biology-related research on terpenoid synthesis.

### Leaves and inflorescences at different developmental stages are important sources of essential oils

We analyzed the metabolome of *O. longilobus* leaves and inflorescences at the exposure, initial opening, and blooming stages. A total of 1,371 highly diverse metabolites were identified. As the inflorescences opened, the amounts and types of metabolites gradually changed. The subsequent classification revealed that terpenoids were the most common metabolites (308 in total), followed by esters, heterocyclic compounds, and other compounds. Among the detected metabolites, terpenoids were the most abundant aromatic substances; these compounds are important for the formation of floral aromas. We speculated that these abundant terpenoids are responsible for the overall strong fragrance of *O. longilobus* plants, which is consistent with the findings of a previous study (Guo et al., 2020).

We detected 293, 302, 293, and 301 terpenoids in the *O. longilobus* leaves and the inflorescences at the exposure, initial opening, and blooming stages, respectively. The comparison of the leaves and inflorescences indicated 11 terpenoids were exclusive to the inflorescences, whereas six terpenoids were specific to the leaves. Thus, although *O. longilobus* leaves contain various terpenoids, the relative abundance of terpenoids appears to be greater in inflorescences. There were four kinds of terpenoids that were specific to the inflorescences at the exposure and blooming stages. However, there were no terpenoids detected only in the inflorescences at the initial opening stage. Accordingly, there were no major changes to the types of terpenoids in the *O. longilobus* inflorescences from the exposure stage to the blooming stage. Certain terpenoids associated with flowers are produced only in specific parts of *O. longilobus* plants and during specific inflorescence developmental stages. Notably, inflorescences can be harvested from the exposure stage to the blooming stage.

On the basis of the relative metabolite contents, terpenoids were more abundant than the other compounds in the leaves and inflorescences of *O. longilobus*. More specifically, the relative contents of the terpenoids in the leaves and the inflorescences at the exposure, initial opening, and blooming stages were 48.57%, 42.27%, 41.41%, and 46.75%, respectively (i.e., higher in the leaves than in the inflorescences).

Compositional analysis of the essential oil extracted from *O. longilobus* leaves (2.07‰ extraction rate for the steam distillation method) identified the three most abundant compounds as terpenoids. According to our previous study, the extraction rate of essential oil from *O. longilobus* inflorescences was 0.43‰ (Liu et al., 2022), which was lower than that from leaves. These results indicate that leaves are important plant tissues for extracting essential oils. Therefore, when developing new varieties, we should consider varieties with fragrant flowers and also choose varieties with fragrant leaves; the essential oil content of leaves was much higher than that of flowers, and leaf essential oil can be extracted over three seasons each year (i.e., spring, summer, and autumn), thus improving the economic value of *O. longilobus*.

Furthermore, we found that the relative content of linalyl acetate peaked in the leaves and inflorescences at the exposure and blooming stages. Earlier research demonstrated that linalyl acetate has a variety of biological effects (e.g., antibacterial and anti-inflammatory effects) (Peana et al., 2002; Kumar et al., 2021). In a previous study, a comparison of the anti-inflammatory properties of linalool and linalyl acetate indicated that at equimolar doses, the effect of linalyl acetate on local edema was less than that of linalool, which significantly decreased the edema in experimental rats at 1 h after the systemic administration (Peana et al., 2002). These results suggest essential oils containing linalool and linalyl acetate may have substantial anti-inflammatory activities. Kumar et al. revealed the significant antifungal and anti-aflatoxin effects of sage essential oil, which may be combined with linalyl acetate (1:1) from herbs to produce a new grass-based anti-inflammatory agent with antifungal, anti-aflatoxin, and antioxidant properties (Kumar et al., 2021). The *Hanyuan zanthoxylum* essential oil, which contains linalyl acetate (14.71%), reportedly has clear inhibitory effects on specific bacteria, especially *Staphylococcus aureus*; however, its effects on *Escherichia coli* are relatively weak (Zhu et al., 2011). Additionally, Guimaraes et al. evaluated the antibacterial activities of 33 terpenoids commonly found in essential oils. Eugenol and carvacrol have good antibacterial effects on *S. aureus*, which is commonly found in food, whereas carveol, citronellol, and geraniol have rapid bactericidal effects on *E. coli*, making them useful for ensuring food safety (Guimaraes et al., 2019). Accordingly, terpenoids have strong inhibitory effects on both bacteria and plant pathogenic fungi. The essential oils extracted from *O. longilobus* leaves and inflorescences may be used as a natural food preservative.

Conclusion

In this study, we analyzed the transcriptomes and metabolomes of the *O. longilobus* leaves, buds, and inflorescences at the exposure, initial opening, and blooming stages. Using a GC-MS platform and a self-built database, a total of 308 terpene metabolites were detected in *O. longilobus* leaves, buds, and inflorescences. The analysis of the terpene metabolic pathway of *O. longilobus* identified 56 candidate genes related to terpene synthesis. The expression of *OlPMK2*, *OlMVK1*, *OlTPS1*, and *OlTPS3* may lead to the accumulation of 11 different terpenoids specifically in the inflorescences at the exposure, initial opening, and blooming stages. The changes in the terpenoid aromatic substances in *O. longilobus* were revealed. We demonstrated that inflorescences can be harvested at the exposure, initial opening, and blooming stages. Moreover, *O. longilobus* leaves could be used as a source of essential oil, thereby increasing the economic value of this plant species. To the best of our knowledge, this study is the first to explore the gene regulatory network related to terpenoids in *O. longilobus* and to identify candidate genes with key regulatory roles. Nevertheless, the data presented herein may be useful for the breeding of new *O. longilobus* varieties and the continued development of the available *O. longilobus* resources, which may ultimately lead to economic benefits.

## Data availability statement

The original contributions presented in the study are publicly available. This data can be found here: NCBI, PRJNA868507.

## Author contributions

YC, HL, HC: Writing–Original Draft & Investigation. HL, WC: Writing–Review & Editing. YL, CL, DC, XC: Investigation. HL, SG, LS, XT: Data curation. WL: Resources. XZ: Project Administration. CH: Supervision. All authors contributed to the article and approved the submitted version.

## Funding

This work was supported by Beijing Innovation Consortium of Agriculture Research System (BAIC09-2022), the Innovation Foundation of the Beijing Academy of Agriculture and Forestry Sciences (KJCX20200112), the Special Fund for Reform and Development, “Evaluation of volatile flavor components in food and establishment of fingerprint” (GGFA292206) and the National Natural Science Foundation of China (31901354). These funding bodies did not help design the study or participate in collecting, analyzing, and interpreting the data or in the writing of the manuscript.

## Acknowledgment

We thank Liwen Bianji, (Edanz) (www.liwenbianji.cn/ac) for editing the English text of a draft of this manuscript.

## Conflict of interest

The authors declare that the research was conducted in the absence of any commercial or financial relationships that could be construed as a potential conflict of interest.

## Publisher’s note

All claims expressed in this article are solely those of the authors and do not necessarily represent those of their affiliated organizations, or those of the publisher, the editors and the reviewers. Any product that may be evaluated in this article, or claim that may be made by its manufacturer, is not guaranteed or endorsed by the publisher.

## Supplementary material

Supplementary File 1 Primer sequences for the qRT-PCR analysis of gene expression.

Supplementary File 2 Summary of all the metabolites detected in *O. longilobus* leaves and inflorescences.

Supplementary File 3 Terpenoid metabolites in *O. longilobus* leaves and inflorescences.

Supplementary File 4 Relative contents of metabolites in *O. longilobus* leaves and inflorescences.

Supplementary File 5 Information regarding the functional annotation of the differentially expressed genes between the buds and inflorescences at the exposure stage.

Supplementary File 6 Information regarding the functional annotation of the differentially expressed genes between the inflorescences at the exposure and initial opening stages.

Supplementary File 7 Information regarding the functional annotation of the differentially expressed genes between the inflorescences at the initial opening and blooming stages.

Supplementary File 8 Information regarding the functional annotation of the differentially expressed genes between the leaves and inflorescences at the initial opening stage.

Supplementary File 9 Genes related to terpene biosynthesis in *O. longilobus*.

Supplementary File 10 All of the metabolites detected in the essential oil extracted from *O. longilobus* leaves.

## Abbreviations

DEG, Differentially expressed gene; DAM, Differentially accumulated metabolite; GC-MS, Gas chromatography-mass spectrometry; HS-SPME, Headspace solid phase microextraction; OPLS-DA, Orthogonal partial least squares discriminant analysis; PCC, Pearson’s correlation coefficient; PCR, Polymerase chain reaction; qRT-PCR, Quantitative real-time PCR.
